# Recurrent Acute Pancreatitis Associated With Hydrochlorothiazide Use: A Case Report

**DOI:** 10.7759/cureus.77108

**Published:** 2025-01-07

**Authors:** Benjamin K Linkous, Angeli J Canekeratne, Mohamed Naas, John G Brunner

**Affiliations:** 1 Medical School, Florida State University College of Medicine, Tallahassee, USA; 2 Internal Medicine, Jackson Hospital, Marianna, USA

**Keywords:** acute pancreatitis, adverse drug reaction, drug-induced acute pancreatitis, medication reconciliation, severe pancreatitis, thiazide-associated pancreatitis, thiazide diuretics

## Abstract

Acute pancreatitis (AP) is a common inflammatory condition of the pancreas, often caused by gallstones or alcohol. However, drug-induced acute pancreatitis (DIAP) is a rare and challenging diagnosis that requires thorough medication reconciliation and a high degree of clinical suspicion. This case report describes a 58-year-old African-American female patient who presented to the emergency department on two occasions with severe epigastric pain, elevated lipase levels, and imaging findings consistent with AP. After excluding common causes of AP, hydrochlorothiazide (HCTZ) was identified as the likely trigger. Discontinuation of HCTZ resulted in symptom resolution, with no recurrence during follow-up. This case underscores the importance of considering DIAP, particularly in patients on HCTZ who present with unexplained, recurrent AP.

## Introduction

Acute pancreatitis (AP) is an inflammatory condition of the pancreas with diverse clinical presentations and significant public health implications, accounting for approximately 200,000-250,000 hospital discharges annually in the United States [1]. While the majority of cases are linked to gallstones or alcohol use, less prevalent causes include hypertriglyceridemia and adverse drug reactions [1]. The Revised Atlanta Classification provides a standardized, international criterion for diagnosing AP, requiring at least two of the following three criteria: (1) serum lipase or amylase levels elevated to three times the upper limit of normal, (2) abdominal pain consistent with pancreatitis, and (3) imaging findings indicative of AP [1]. Drug-induced acute pancreatitis (DIAP), although rare, accounts for up to 2% of all AP cases and is particularly important in recurrent or idiopathic presentations [2]. DIAP was first described in 1959 by Johnston and Cornish in association with thiazide diuretics in three patients [3]. A literature review using PubMed and keywords such as "thiazide-induced pancreatitis" and "thiazide diuretics" identified fewer than 20 published cases, with this case contributing to the existing literature [2-9]. Over 200 medications are now implicated in DIAP, with hydrochlorothiazide (HCTZ) being a notable association [2,4]. The mechanisms through which HCTZ induces pancreatitis remain unclear, but proposed mechanisms include direct toxicity to pancreatic acinar cells, disturbances in electrolyte and fluid balance, and drug-induced hypercalcemia exacerbating pancreatic inflammation [4]. 

Here, we present the case of a 58-year-old African-American female patient who developed recurrent AP while on HCTZ therapy, further contributing to the limited body of literature on this topic. While rare, this case underscores the importance of considering medication history in the evaluation of patients with recurrent or idiopathic pancreatitis. 

## Case presentation

First hospitalization

A 58-year-old African-American female patient presented to the emergency department on the evening of January 14, 2023, with a four-day history of persistent epigastric pain. She described the pain as gnawing and unrelenting, without associated nausea, vomiting, diarrhea, or constipation. The symptoms began shortly after consuming a chili cheese dog. Her past medical history included poorly controlled insulin-dependent type 2 diabetes mellitus (16 years), chronic kidney disease stage IIIb (6 years), and recurrent pancreatitis of unclear etiology over the past two years. She denied recent alcohol use, and her social history was negative for smoking or illicit drug use.

**Figure 1:**
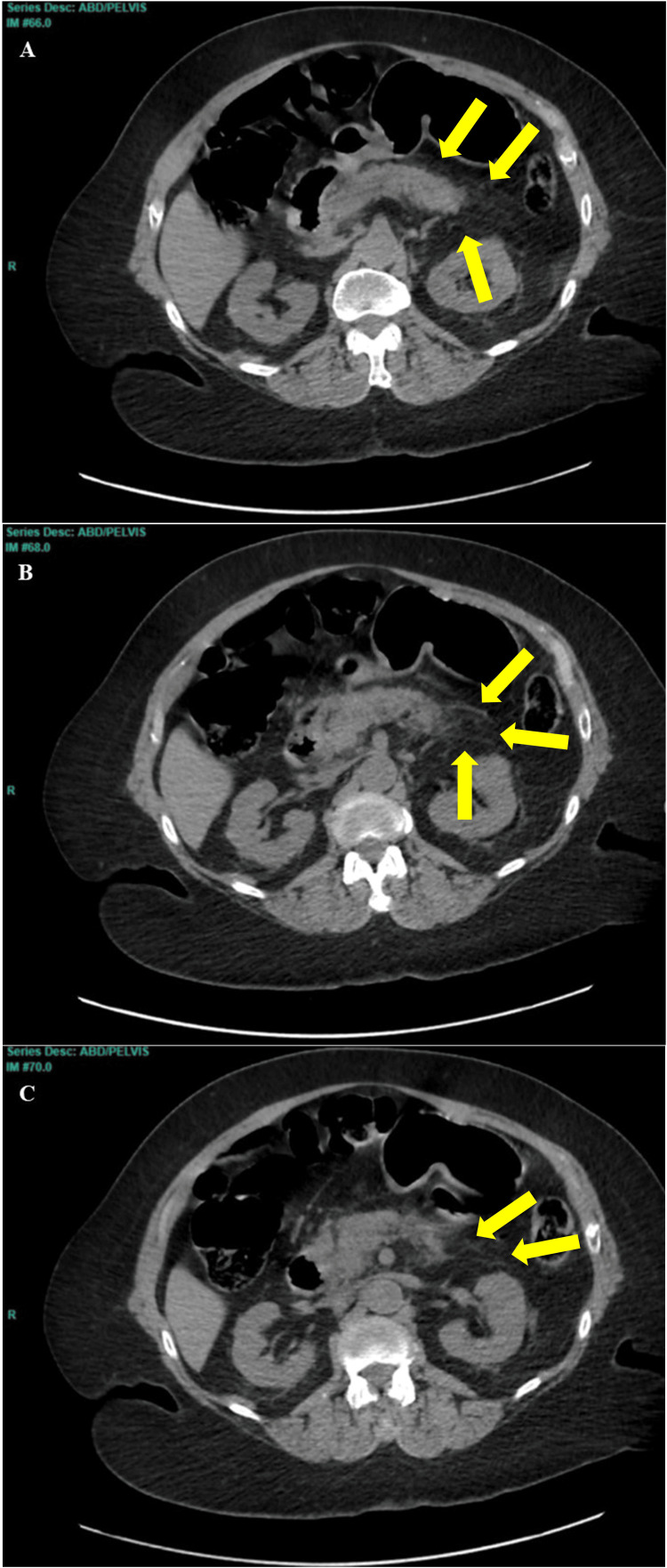
(A-C) Computed tomography of the abdomen and pelvis without intravenous contrast The images show acute interstitial pancreatitis with peripancreatic inflammatory stranding (yellow arrows) and no focal fluid collection. The images are presented in a superior-to-inferior orientation: (A) most superior and (C) most inferior.

Initial vital signs showed mild tachycardia, but the patient was otherwise hemodynamically stable. Laboratory results revealed acute kidney injury, hypokalemia, hyperglycemia, elevated lipase, and elevated triglycerides. The white blood cell count was also elevated, suggesting inflammation (Table 1). CT imaging of the abdomen and pelvis revealed peripancreatic inflammatory stranding around the pancreas, consistent with AP, without evidence of gallstones, ductal obstruction, or pseudocysts (Figures 1A-1C).

The patient met all three of the Revised Atlanta Criteria for AP and was subsequently admitted for aggressive intravenous fluid resuscitation, pain management with morphine, and bowel rest. A low-fat diet was introduced as her symptoms improved. Given her history of diabetes mellitus and the potential for medication-induced pancreatitis, HCTZ was considered a potential trigger. Although rare, HCTZ-induced pancreatitis was deemed plausible in this context. Diabetic medications with known associations with pancreatitis, such as semaglutide and other glucagon-like peptide-1 agonists, were discontinued prior to hospitalization. An alternative antihypertensive regimen of oral amlodipine besylate 10 mg daily was started.

Her hospital course was complicated by transient anemia, with hemoglobin gradually decreasing until it reached its lowest level on hospital day 4, requiring iron supplementation which subsequently stabilized hemoglobin levels (Table 1). By January 20, 2023, the patient reported significant improvement in her symptoms, and repeat imaging showed resolution of peripancreatic inflammation. She was discharged in stable condition with a plan to discontinue HCTZ and follow up outpatient for further management of her recurrent pancreatitis.

**Table 1:** Lab test results on the first hospitalization The table presents key lab parameters (e.g., complete blood count, kidney function, electrolytes, lipid profile, and glucose levels) during the patient's first hospitalization. Reference ranges are listed for comparison to aid interpretation. Upwards arrow (↑), above reference range; downwards arrow (↓), below reference range; and hyphen (-), not available. WBC: white blood cell count, RBC: red blood cell count, HGB: hemoglobin, HCT: hematocrit, MCV: mean corpuscular volume, MCH: mean corpuscular hemoglobin, MCHC: mean corpuscular hemoglobin concentration, RDW: red blood cell distribution width, MPV: mean platelet volume, PLT: platelet, TIBC: total iron-binding capacity, UIBC: unsaturated iron-binding capacity, %SAT: percent iron saturation, BUN: blood urea nitrogen, HDL: high-density lipoprotein, LDL: low-density lipoprotein, GFR: glomerular filtration rate, AA: African American.

Lab test	Reference range	Day 1 (01/14/2023)	Day 2 (01/15/2023)	Day 3 (01/16/2023)	Day 4 (01/17/2023)	Day 5 (01/18/2023)	Day 6 (01/19/2023)	Day 7 (01/20/2023)
WBC	3.8-11.1 x 10^3^/uL	11.3 ↑	11.6 ↑	6.6	4.9	5.3	-	-
RBC	3.64-5.41 x 10^6^/uL	4.76	4.13	3.12 ↓	2.93 ↓	3.28 ↓	-	-
HGB	11.1-15.8 g/dL	14.1	12.4	9.4 ↓	8.8 ↓	9.9 ↓	-	-
HCT	34.3%-46.7 %	42.0	36.2	29.4 ↓	28.4 ↓	30.9 ↓	-	-
MCV	80.1-99.4 fL	88.2	87.7	94.2	96.9	94.2	-	-
MCH	26.4-33.5 pg	29.6	30.0	30.1	30.0	30.2	-	-
MCHC	31.1-35.5 g/dL	33.6	34.3	32.0	31.0 ↓	32.0	-	-
RDW	36-50 fL	42	42.0	46.0	48.0	48.0	-	-
MPV	9.4-12.3 fL	13.1 ↑	13.2 ↑	13.3 ↑	12.5 ↑	12.4 ↑	-	-
PLT	139-391 x 10^3^/uL	372	242	202	158	210	-	-
Iron	35-150 ug/dL	-	-	-	<37 ↓	-	-	-
TIBC	260-445 ug/dL	-	-	-	223 ↓	-	-	-
UIBC	130-300 ug/dL	-	-	-	187	-	-	-
%SAT	18%-48%	-	-	-	16 ↓	-	-	-
Glucose	70-99 mg/dL	-	138 ↑	148 ↑	146 ↑	-	347 ↑	-
BUN	7-18 mg/dL	26 ↑	31 ↑	27 ↑	19 ↑	-	-	-
Creatinine	0.6-1.3 mg/dL	2.3 ↑	2.7 ↑	2.4 ↑	1.9 ↑	-	1.7 ↑	-
BUN/creatinine	5-20	11.2	12.0	-	-	-	-	-
Sodium	135-145 mmol/L	131 ↓	139	138	139	-	140	-
Potassium	3.5-5.0 mmol/L	4.0	3.3 ↓	3.7	3.6	-	4.1	-
Chloride	98-109 mmol/L	93 ↓	102	107	110 ↑	-	109	-
CO2	17.0-34.0 mmol/L	21.0 ↓	23.5	19.7 ↓	17.6 ↓	-	21.6	-
Anion gap	3.0-18.0 mmol/L	21.0 ↑	16.8	15.0	15.0	-	13.5	-
Calcium	8.5-10.1 mg/dL	-	9.2	7.9 ↓	7.6 ↓	-	8.4 ↓	-
Cholesterol	50-170 mg/dL	-	131	-	-	-	-	-
Triglycerides	30-200 mg/dL	-	323 ↑	-	-	-	-	-
HDL	40-83 mg/dL	-	20 ↓	-	-	-	-	-
LDL	0-129 mg/dL	-	46	-	-	-	-	-
Chol/HDL ratio	0.0-5.0	-	6.6 ↑	-	-	-	-	-
GFR AA	>60	26 ↓	22 ↓	25 ↓	33 ↓	-	37 ↓	-
GFR non-AA	>60	22 ↓	18 ↓	21 ↓	27 ↓	-	31 ↓	-
Lipase	73-393 U/L	6968 ↑	-	-	-	-	-	-
Lactate	0.7-2.0 mmol/L	2.7 ↑	2.4 ↑	-	-	-	-	-

Second hospitalization

Sixteen months later, on June 3, 2024, the now 59-year-old patient presented to the emergency department with recurrent abdominal pain, nausea, and vomiting. She noted that the pain had gradually worsened over several days and resembled her prior episodes of pancreatitis. Despite previous recommendations to discontinue HCTZ, a medication reconciliation revealed that she had resumed HCTZ use approximately 1-2 weeks prior to her second hospitalization, without consulting her healthcare providers. She denied alcohol use, recent dietary triggers, or changes in her medication regimen, aside from the reintroduction of oral HCTZ 25 mg daily.

On physical examination, the patient appeared well-nourished but exhibited tenderness in the epigastric and right upper quadrant regions. Laboratory studies were notable for elevated lipase, hypercalcemia, hypokalemia, and hyperglycemia (Table 2). CT imaging confirmed peripancreatic inflammation consistent with AP and no evidence of gallbladder pathology or ductal obstruction.

**Table 2:** Lab test results on the second hospitalization The table presents key lab parameters (e.g., complete blood count, kidney function, electrolytes, and glucose levels) during the patient's second hospitalization. Reference ranges are listed for comparison to aid interpretation. Upwards arrow (↑), above reference range; downwards arrow (↓), below reference range; and hyphen (-), not available. WBC: white blood cell count, RBC: red blood cell count, HGB: hemoglobin, HCT: hematocrit, MCV: mean corpuscular volume, MCH: mean corpuscular hemoglobin, MCHC: mean corpuscular hemoglobin concentration, RDW: red blood cell distribution width, MPV: mean platelet volume, PLT: platelet, BUN: blood urea nitrogen, GFR: glomerular filtration rate, AA: African American.

Lab test	Reference range	Day 1 (06/03/2024)	Day 2 (06/04/2024)	Day 3 (06/05/2024)	Day 4 (06/06/2024)	Day 5 (06/07/2024)
WBC	3.8-11.1 x 10^3^/uL	7.9	-	-	-	6.1
RBC	3.64-5.41 x 10^6^/uL	4.44	-	-	-	4.19
HGB	11.1-15.8 g/dL	13.9	-	-	-	13.0
HCT	34.3-46.7%	42.3	-	-	-	40.3
MCV	80.1-99.4 fL	95.3	-	-	-	96.2
MCH	26.4-33.5 pg	31.3	-	-	-	31.0
MCHC	31.1-35.5 g/dL	32.9	-	-	-	32.3
RDW	36-50 fL	49.0	-	-	-	48.0
MPV	9.4-12.3 fL	11.4	-	-	-	12.2
PLT	139-391 x 10^3^/uL	266	-	-	-	202
Glucose	70-99 mg/dL	390 ↑	279 ↑	257 ↑	-	296 ↑
BUN	7-18 mg/dL	23 ↑	19 ↑	17	-	15
Creatinine	0.6-1.3 mg/dL	2.0 ↑	1.8 ↑	1.7 ↑	-	1.5 ↑
BUN/creatinine	5-20	11.0	11.0	10	-	10
Sodium	135-145 mmol/L	132 ↓	133	138	-	139
Potassium	3.5-5.0 mmol/L	3.2	2.9 ↓	3.2	-	3.2
Chloride	98-109 mmol/L	92 ↓	95 ↓	97 ↓	-	102
CO2	17.0-34.0 mmol/L	26.5	22.7	27.0	-	27.9
Anion gap	3.0-18.0 mmol/L	13.5	15.3	14.0	-	9.1
Calcium	8.5-10.1 mg/dL	10.6 ↑	10.1	9.6	-	9.1
Triglycerides	30-200 mg/dL	152	-	-	-	-
GFR AA	>60	28 ↓	32 ↓	34 ↓	-	40 ↓
Lipase	16-77 U/L	142 ↑	-	-	-	-
Lactate	0.7-2.0 mmol/L	2.0	-	-	-	-

The patient met two of the three Revised Atlanta Criteria and was admitted for management with aggressive intravenous fluids, opioid analgesia, and antiemetic therapy. Despite initial interventions, her nausea persisted, necessitating the introduction of a scheduled Reglan (metoclopramide) 10 mg injection every six hours. Hypercalcemia was managed with fluid resuscitation, and hypokalemia was corrected with intravenous potassium supplementation.

Over the next five days, her abdominal pain gradually improved, and she tolerated a soft, low-fat diet. The persistent nausea was attributed to suspected diabetic gastroparesis, for which Reglan (metoclopramide) was continued. The re-initiation of HCTZ was strongly implicated as the cause of her recurrent pancreatitis, given the temporal relationship and the exclusion of other common etiologies. At discharge on June 8, 2024, the patient was instructed to permanently discontinue HCTZ and to follow a bland diabetic diet. She was prescribed Reglan (metoclopramide) 10 mg orally every eight hours as needed and Zofran (ondansetron) 4 mg orally every eight hours as needed to manage her gastrointestinal symptoms. She was also advised to follow up with her primary care physician for long-term management.

## Discussion

This case highlights a temporal association between HCTZ use and recurrent episodes of AP in a patient without other identifiable triggers. During the first hospitalization, the etiology of pancreatitis was ambiguous, with negative imaging findings for gallstones and an unremarkable alcohol history. Given the patient's triglyceride level of 323 mg/dL, hypertriglyceridemia was also considered a potential cause for the first episode of pancreatitis, as it is the third most common cause of AP. However, elevations in triglyceride levels below 500 are rarely associated with AP in the literature, with levels greater than 1000 generally required to trigger the condition [10]. While there is no standardized threshold for hypertriglyceridemia-associated pancreatitis, the normal triglyceride level during the second admission further supports that hypertriglyceridemia was an unlikely cause of her recurrent episodes.

The patient’s continued use of HCTZ raised suspicion, supported by case reports in the literature linking thiazide exposure to pancreatitis [11]. After discontinuation of HCTZ, the patient experienced clinical improvement and has not had any additional pancreatitis hospitalizations as of this report’s submission, further suggesting a potential causal relationship.

The recurrence of acute pancreatitis during the second hospitalization following HCTZ re-initiation strengthens the likelihood of causation. Notably, the patient’s laboratory findings during the second episode, including hypercalcemia, align with proposed mechanisms of HCTZ-induced pancreatic injury. Furthermore, this pattern aligns with published data, such as studies by Bellochi et al., which utilized the Naranjo Scale to categorize HCTZ as a definite cause of DIAP, particularly in the presence of hypercalcemia or dehydration [2,12]. The Naranjo Scale evaluates causality based on parameters like the temporal relationship between drug exposure and symptoms, improvement upon drug discontinuation, and recurrence upon rechallenge [12].

Thiazide-associated pancreatitis is classified as a rare and idiosyncratic reaction, often diagnosed by exclusion. Despite its rarity, clinicians should maintain a high index of suspicion when evaluating patients with unexplained recurrent pancreatitis who are on thiazide diuretics. This case also underscores the importance of meticulous medication reconciliation and patient education, as inadvertent re-exposure to the offending agent can lead to preventable morbidity.

While the temporal association and absence of alternative explanations strongly suggest HCTZ-induced pancreatitis in this patient, limitations exist. Applying the WHO-UMC causality assessment scale, this case would likely be categorized as "probable" or "likely," given the clear temporal relationship and absence of confounding factors [13,14]. However, the absence of rechallenge data, a gold standard for confirming drug causality, is a significant barrier to establishing "certain" causality [13-15]. The ethical and clinical risks associated with intentional rechallenge preclude this approach in practice. Future studies with pharmacovigilance data or prospective designs are needed to better characterize the mechanisms and risk factors for thiazide-associated pancreatitis.

## Conclusions

This case underscores the significance of recognizing thiazide diuretics, particularly HCTZ, as a potential cause of AP. In patients presenting with recurrent pancreatitis of unclear etiology, a thorough review of medication history is essential. Discontinuation of HCTZ in this patient led to symptom resolution, reinforcing the need for vigilance in medication management.

The findings of this case align with existing literature, suggesting that thiazide-associated pancreatitis, though rare, should be considered in patients on thiazides, particularly in the presence of hypercalcemia or recurrent idiopathic pancreatitis. Avoidance of rechallenge and selection of alternative antihypertensive therapies are critical to reducing recurrence risk. Further research is needed to better understand the pathophysiological mechanisms underlying thiazide-associated pancreatitis and to identify patient populations at higher risk for this rare but significant adverse drug reaction.
